# Mutivariate optimization strategy for the sonication-based extraction of *Nardostachys jatamansi* roots and analysis for chemical composition, anti-oxidant and acetylcholinesterase inhibitory potential

**DOI:** 10.1016/j.ultsonch.2022.106133

**Published:** 2022-08-24

**Authors:** Ashwani Arya, Vineet Mittal, Deepak Kaushik, Manish Kumar, Saqer S. Alotaibi, Sarah M. Albogami, Gaber El-Saber Batiha, Philippe Jeandet

**Affiliations:** a Department of Pharmaceutical Sciences, Maharshi Dayanand University, Rohtak 124001, Haryana, India; b M.M. College of Pharmacy, Maharishi Markandeshwar (Deemed to be University), Mullana (133207), Ambala, Haryana, India; c Department of Biotechnology, College of Science, Taif University, P.O. Box 11099, Taif, Saudi Arabia; d Department of Pharmacology and Therapeutics, Faculty of Veterinary Medicine, Damanhour University, Damanhour, AlBeheira, Egypt; e University of Reims, Research Unit-Induced Resistance and Plant Bioprotection, EA 4707 - USC INRAe 1488, SFR Condorcet FR CNRS 3417, 51687 Reims, France

## Abstract

Extracts from medicinal plants are generally obtained by conventional methods like percolation and maceration. Owing to limitations of traditional methods and to meet the rising demand of extracts, the development of new green approaches is need of hour. In the present research, we have developed an ultrasound-assisted extraction (UAE) method for the *Nardostachys jatamansi* (NJ) D. Don, DC roots and optimized the extraction parameters for possible improved extract yield. A multivariate optimization strategy using the Centre Composite Design coupled with response surface methodology was applied. A numerical optimization approach accurately predicted the extraction conditions (sonication time ∼ 20 min, ethanol ∼ 70 % and a liquid/solid ratio of about 21:1). Scanning electron microscopy of the plant samples after UAE also indicated the cavitation effect due to sound waves. GC–MS analysis of the optimized ultrasound extract (OUNJ) confirmed improvement in the concentration of various secondary metabolites like jatamansone (91.8 % increase), spirojatamol (42.3 % increase), globulol (130.4 % increase), sitosterol (84.6 % increase) as compared to the soxhlet extract (SXNJ). Different anti-oxidant parameters (DPPH, Glutathione, Catalase SOD and NO) were also significantly altered (p < 0.05) in the optimized extracts. The IC_50_ to inhibit acetylcholinesterase activity (AChE) *in vitro* and its concentration in brain homogenates were significantly (p < 0.05) improved by OUNJ extract as compared to the SXNJ ones. To conclude, we can say that established optimized conditions for UAE of *N. jatamansi* roots not only reduce the extraction time but also improved the pharmacological potential of the extracts.

## Highlights

• Multivariate optimization strategy predicted the ultrasound-assisted extraction (UAE) conditions for *Nardostachys jatamansi* root extracts.
• GC–MS analysis pointed out improvement in the concentration of the bioactive sesquiterpenes and steroidal compounds with the UAE method.
• Optimized extracts using the UAE method demonstrated better anti-oxidant and AChE inhibitory potentials.
• UAE could be assumed as a ‘green approach’ for the extraction of selected medicinal herbs.

## Graphical abstract

**Figure:**
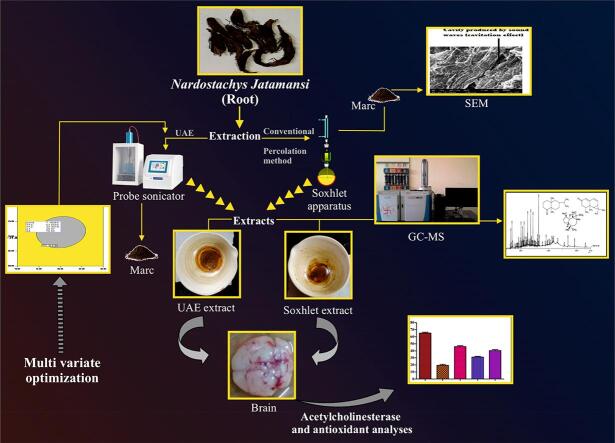

## 1 Introduction

There is a considerable demand for extracts of herbs by the different manufacturing or cosmetic units in India and different regions of world. Also, the plant-based formulations are fetching more attention from people all around the world, as these products are considered more effective, cheaper, and with lesser side effects. Globally, the commercial significance of herbal formulations/products is growing steadily, as well. Moreover, it is also expected that worldwide trade of such products could go above 100 billion USD in coming years. Traditional techniques of extraction constitute very time-consuming processes and are not considered to be environment friendly and it would be harsh to rely only on conventional extraction methods to deal with such demands. Hence to meet the rising demand of extracts and to maintain their sustainable supply, the application of unorthodox techniques of extraction is need of hour. Thus, unconventional methods such as ultrasound-assisted extraction (UAE), supercritical fluid extraction (SFE) and pressurized liquid extraction (PLE) should be addressed [1]. These processes are not only fast but could also improve the yields of extracts/secondary metabolites and could prove as an alternative for the industry to get the herbal extracts in a sustainable, economical and greener manner [2].

In the last decade, UAE has been often employed for the extraction of medicinal plants [3], [4]. In this technique, the energy of sound waves above the 20 KHz frequency produced the acoustic cavitation effect in the particles of the crude drug to bring out the solubilized extract. Channels are created in the herb particles due to strong shear forces generated by the bursting of bubbles near the surface of the plant material. These channels help to the diffusion of the solvents more efficiently inside the plant matrix and improve the mass transfer, which leads to enhanced yields of extracts and secondary metabolites [5], [6]. In the past, various researchers have utilized this technique for the enhanced recovery of plant bioactive components/extracts in a fast, cost-effective and environmentally friendly approach [2], [5].

The multivariate optimization strategies could statistically collate the effects of different variables in a complex system to get the desired results [7], [8]. The influencing factors in UAE like sonication power, solvent to solute ratio, time of extraction, choice of solvent, pH etc, which extensively affect the complexity of the extraction process, could be optimized by the application of design approaches for the accuracy and reliability of the data. In the recent years, many optimization approaches have been mingled in the UAE process to get the optimum conditions for extraction of herbs [2]. Moreover, the response surface methodology (RSM) coupled with different optimization design has proven to be precise in determining the optimum region as compared to single factor analysis (SFA) [9].

The selected medicinal herb, *Nardostachys jatamansi* (NJ) D.Don, DC (Family- Caprifoliaceae), is also a highly traded medicinal plant in India with significant therapeutic potential [10], [11]. Generally, it is collected from wild sources in alpine regions of the Himalayas from Himachal Pradesh to Sikkim and Bhutan at an altitude of 3000–5000 m. The herb, *Jatamansi* or Indian spikenard, is mentioned in ‘*Sushrut Samhita’*, ‘*Charak Samhita’* and various other ayurvedic literatures, for preventing mental disorders such as epilepsy, anxiety, depression, etc [12], [13]. It is also highly mentioned in the traditional literature for improvement in cognition, alertness and concentration [14]. Various pharmacological reports have also supported the anti-oxidant, learning and memory enhancement, CNS-protective, anti-anxiety, anticonvulsant, antidepressant, anti-inflammatory and anti-Parkinson’s potentials of selected herbs [15], [16], [17], [18]. The potent pharmacological importance of *Jatamansi* fetches the attention of industries and numerous commercial formulations of this plant such as ‘Mentat’ ‘Mentat DS’ (Himalaya Herbal Healthcare, India), ‘Intellimax’ (Piramal Phytocare, India) are available in the market for improvement of the brain health. Besides the improvement in CNS disorders, this herb is also used commercially for the management of skin disorders and hair fall [19].

Owing to the pharmacological importance and industrial prospects of *N. jatamansi*, this plant was selected for the present study. It is aimed to extract the herb by an un-conventional method, UAE and to optimize the various extraction parameters for possible improvement in the yields of plant actives/extracts. Scanning Electron Microscopy (SEM) was also performed to confirm the mechanical effects of sound waves on plant samples. On the other hand, the chemical composition of the extracts was analyzed using the gas chromatography coupled mass spectroscopy (GC–MS) technique. As the therapeutic potential, especially the nootropic effect of *N. jatamansi* is mainly due to its anti-oxidant and anti-cholinesterase properties, the different extracts were also evaluated for their anti-oxidant and acetylcholinesterase (AChE) inhibitory potential.

## 2 Materials and methods

### 2.1 Plant samples and reagents

The roots of *N. jatamansi* were procured from a local market in Hisar, Haryana and authenticated by an eminent botanist. The roots were processed, pulverized and sieved (size 40) to get the coarse powder of the herb. The powdered herb was kept in air-tight containers till use. The solvents employed in the extraction process were of analytical grade. Scopolamine and donepezil were purchased from Sigma Aldrich, USA.

### 2.2 Extraction of the selected herb

The root powder of the plant was extracted using different extraction methods. Generally, the selected plant, *N. jatamansi*, is extracted by conventional methods such as the maceration and percolation ones. In the present study, plant samples were extracted by the conventional percolation method (CPM) using soxhlet apparatus. For this, the powder sample (10 g) was filled in the extraction chamber and the extraction was carried out using ethanol as solvent. The process was continued till exhaustion of the extraction process [20], [21]. The powdered herb was also extracted by a non-conventional green technique such as UAE. The probe ultrasonicator (UP-800, Chrome Tech. Co, ltd, Taiwan) was used for the extraction by sound waves. The apparatus was equipped with a sound abating enclosure along with a probe (Ø −5/8″) and a temperature control sensor. The extraction was carried out at different experimental conditions as suggested by the design software at a fixed frequency of 24 KHz, higher intensity and with a pulse of 0.5 *sec*. During the process, the tip of the ultrasonic probe was dipped to 1cm-height in the solvent [22]. After the extraction process using the two different methods, the solution was centrifuged for 10 min at 5000 rpm and filtered through Whatman filter paper. The filtrate was concentrated to get the solid extract and the percentage yield (w/w) was determined. The extract was kept in an air-tight container till next experiments.

### 2.3 Experimental design

The various process variables like the sonication time (X_1_), the solvent concentration (X_2_) and the volume of the solvent (X_3_) were optimized for extract yield, EY, (Y_1_), total phenolic content (TPC, Y_2_) and total flavonoid content (TFC, Y_3_) in the sonication-based extraction of the herb. The optimization range of the various independent variables is given in Table 1. The central composite design (CCD) was employed, which suggested the various experimental conditions (20 runs) to carry out the extraction process in UAE (see Table 2). The extract yield, TPC and TFC of the different runs were also determined by specified procedures. The linear (*β_i_*), quadratic (*β_ii_*) and interactive (*β_ij_*) terms for different independent variables, indicating their effect on various responses (Y) along with the coefficient of interception (*β_0_*) and error (*ε*) were also represented by a standard polynomial equation (eq. 1).

(1) $$\left(Y\right)=\beta_{0}+\sum_{i=1}^{n}\beta_{i}x_{i}+\sum_{i=1}^{n}\beta_{\mathrm{ii}}x_{i}^{2}+\sum_{i\neq j}^{n}\beta_{\mathrm{ij}}x_{i}x_{j}+\varepsilon$$

**Table 1:** Actual and predicted extraction yields, TPC and TFC at different extraction conditions in UAE.

**Std**	**Run**	**Sonication time** **(min)** **(X_1_)**	**Solvent Conc. (%)** **(X_2_)**	**Volume of solvent (mL)** **(X_3_)**	**EY** **(%, w/w)**		**TPC** **mg of GAE/g** **of extract**		**TFC** **(mg of RUE/g of extract)**	
					**Actual yield (w/w)**	**Predicted yield (w/w)**	**Actual yield (w/w)**	**Predicted yield (w/w)**	**Actual yield (w/w)**	**Predicted yield (w/w)**
8	1	30.00	80.00	25.00	3.9	4.11	39.34	39.01	17.8	17.69
1	2	10.00	60.00	15.00	4.10	3.68	28.9	28.68	10.8	10.37
2	3	30.00	60.00	15.00	1.70	1.61	18.01	17.48	10.1	9.66
9	4	3.18	70.00	20.00	2.2	2.57	30	30.27	12.1	12.08
18	5	20.00	70.00	20.00	7.2	7.19	52.60	52.44	33.7	33.98
6	6	30.00	60.00	25.00	3.9	3.77	35.90	35.45	18.00	17.40
16	7	20.00	70.00	20.00	7.00	7.19	52.9	52.44	34.00	33.98
4	8	30.00	80.00	15.00	4.10	4.05	35.2	35.22	14.8	14.21
10	9	36.82	70.00	20.00	2.01	1.95	27.1	27.60	12.3	13.08
14	10	20.00	70.00	28.41	6.2	6.22	50.8	51.35	29.2	29.39
3	11	10.00	80.00	15.00	4.90	4.82	39.5	39.40	13.10	13.17
7	12	10.00	80.00	25.00	2.9	2.78	31	30.98	15.9	15.80
17	13	20.00	70.00	20.00	7.2	7.19	51.9	52.44	34.20	33.98
13	14	20.00	70.00	11.59	5.84	6.12	43.1	43.32	20.10	20.67
12	15	20.00	86.82	20.00	3.6	3.52	30.9	30.89	10.2	10.38
19	16	20.00	70.00	20.00	7.4	7.19	52.30	52.44	33.8	33.98
11	17	20.00	53.18	20.00	1.9	2.28	18.1	18.88	7.20	7.78
20	18	20.00	70.00	20.00	7.4	7.19	52.5	52.44	34.5	33.98
5	19	10.00	60.00	25.00	3.90	3.74	35.00	34.44	17.2	17.26
15	20	20.00	70.00	20.00	7.00	7.19	52.6	52.44	33.8	33.98

**Table 2:** ANOVA applied on the results obtained for EY, TPC and TFC in UAE of NJ extracts.

**EY**							**TPC**						**TFC**					
**Source**	**Sum of Squares**	**Df**	**Mean**	**F**	**p-value**		**Sum of Squares**	**Df**	**Mean**	**F**	**p-value**		**Sum of Squares**	**Df**	**Mean**	**F**	**p-value**	
**Model**	77.84	9	8.65	104.91	< 0.0001	Sig.	2613.66	9	290.41	1027.34	< 0.0001	Sig.	1922.38	9	213.60	734.64	< 0.0001	Sig.
**X_1_**	*0.46*	*1*	*0.46*	*5.64*	*0.0390*		*8.58*	*1*	*8.58*	*30.37*	*0.0003*		*1.19*	*1*	*1.19*	*4.10*	*0.0703*	
**X_2_**	*1.87*	*1*	*1.87*	*22.73*	*0.0008*		*174.07*	*1*	*174.07*	*615.78*	*< 0.0001*		*8.14*	*1*	*8.14*	*28.01*	*0.0004*	
**X_3_**	*0.012*	*1*	*0.012*	*0.15*	*0.7104*		*77.72*	*1*	*77.72*	*274.95*	*< 0.0001*		*91.78*	*1*	*91.78*	*315.67*	*< 0.0001*	
**X_1_ X_2_**	*0.85*	*1*	*0.85*	*10.25*	*0.0095*		*24.61*	*1*	*24.61*	*87.04*	*< 0.0001*		*1.53*	*1*	*1.53*	*5.27*	*0.0446*	
**X_1_X_3_**	*2.21*	*1*	*2.21*	*26.75*	*0.0004*		*74.60*	*1*	*74.60*	*263.91*	*< 0.0001*		*0.36*	*1*	*0.36*	*1.24*	*0.2911*	
**X_2_ X_3_**	*2.20*	*1*	*2.20*	*26.75*	*0.0004*		*100.47*	*1*	*100.47*	*355.40*	*< 0.0001*		*9.03*	*1*	*9.03*	*31.06*	*0.0002*	
**X_1_^2^**	*43.88*	*1*	*43.88*	*532.36*	*< 0.0001*		*995.56*	*1*	*995.56*	*3521.89*	*< 0.0001*		*824.95*	*1*	*824.95*	*2837.30*	*< 0.0001*	
**X_2_^2^**	*33.16*	*1*	*33.16*	*402.31*	*< 0.0001*		*1368.14*	*1*	*1368.14*	*4839.89*	*< 0.0001*		*1116.87*	*1*	*1116.87*	*3841.30*	*< 0.0001*	
**X_3_^2^**	*1.88*	*1*	*1.88*	*22.77*	*0.0008*		*47.01*	*1*	*47.01*	*166.32*	*< 0.0001*		*144.29*	*1*	*144.29*	*496.26*	*< 0.0001*	
**Residual**	0.82	10	0.082				2.83	10	0.28				2.91	10	0.29			
**Lack of Adjustment**	*0.66*	*5*	*0.13*	*4.15*	*0.0721*	Not Sig.	*2.25*	*5*	*0.45*	*3.93*	*0.0797*	Not Sig.	*2.45*	*5*	*0.49*	*5.32*	*0.0452*	Not Sig.
**Pure Error**	0.16	5	0.032				*0.57*	*5*	*0.11*				0.46	5	0.092			
**C.V. %**	6.09						1.37						2.61					
**PRESS**	5.34						18.01						19.52					
**R^2^**	0.9895						0.9989						0.9985					
**Adjusted R^2^**	0.9801						0.9979						0.9971					
**Predicted R^2^**	0.9322						0.9931						0.9899					
**Adequate Precision**	27.497						92.993						68.711					

The design expert software (7.0.3, Statease Inc, Minneapolis, USA) was used to determine the significance of the obtained results, the applied model and to get the diagnostic curves [23].

### 2.4 Analysis of TPC and TFC

The TPC and TFC of the various samples were determined by a previously established method [18]. The Folin-Ciocalteu (FC) reagent was used to determine the phenolic content and was expressed as mg of gallic acid equivalent, GAE/g of extracts. Briefly, the various extracts (0.5 g) were dissolved in methanol (10 mL) and the FC reagent (1.5 mL) was added to the solution. The solution was stirred continuously to mix the aqueous Na_2_CO_3_ (1.5 mL) for 10 min. Later, this solution was incubated at 45 °C on a water bath for 30 min. Likewise, for the TFC, the extract solution was prepared in methanol (10 mg/mL) and aqueous aluminum chloride was added to this solution. The mixture was incubated at room temperature for 1 h. Finally, the absorbance of the samples was noted at λ_max_ of 760 and 415 nm, for TPC and TFC, respectively, using a UV spectrophotometer (UV-1800, Shimadzu Scientific Instruments Private Limited). The TFC was expressed as mg of rutin equivalent, RUE/g of extract.

### 2.5 SEM analysis

The powder (untreated) and the marc of the plant samples following the different extraction processes were prepared for scanning electron microscopy. For this, the herb powder was dried for at least two hours under vacuum between 40°-50 °C. The samples were sputtered, coated with gold and scanned at higher vacuum mode for any structural changes under an electron microscope (Zeiss EVO40) [24], [25].

### 2.6 GC–MS analysis of extracts

Separation and identification of the various constituents of the different extracts were carried out using GC–MS analysis (Shimadzu QP-2010 with Thermal Desorption System TD 20 equipped with MS capillary column). A previously reported method was employed to analyze the NJ extracts with minor modifications [26]. Briefly, the sample (1 µL) was injected in split mode and electronic ionization operating at 70 eV. Helium (He) was used as carrier gas (1.5 mL/min) and injector temperature was kept at 260 °C. Initially, the oven temperature was kept at 60 °C for 5 min and increased to 150 °C at a rate of 3 °C/min and held for 10 min. Finally, the temperature was enhanced up to 250 °C with an increment of 4 °C/min. MS chromatograms of the extracts were recorded and the retention time (R_T_) and the percentage area (%) of the different compounds were noted.

### 2.7 Evaluation of the biological activity of the different extracts

Extracts of the selected herb are highly utilized commercially for the development of Ayurvedic formulations in various disorders. Hence, the extracts of the *N. jatamansi* roots obtained by the conventional percolation method (SXNJ) and the sonication technique, at optimized conditions (OUNJ) were evaluated for their *in vitro* and *in vivo* antioxidant potential. The marketed formulations of the NJ extracts are also used for cognition improvement. Therefore, the resulting extracts were tested for their AChE inhibitory potential, as well.

#### 2.7.1 In vitro anti-oxidant assay

The *in-vitro* anti-oxidant potential of the extracts can be determined by analyzing the inhibitory concentration (IC) needed to scavenge the free radicals of 2,2-Diphenyl Picrylhydrazyl (DPPH) [27]. For the assay, the test solution was prepared by mixing DPPH and the extract/standard (0.2 mL). The solution was incubated at room temperature for 30 min and absorbance was measured in an UV–Visible spectrophotometer (λ_max_ 517 nm). The percentage (%) inhibition of DPPH was calculated by measuring the absorbance of the solution (t_0_) and after 30 min (t_30_). The various concentrations of the test samples were prepared to determine the IC_50_ against fifty percent of free radicals of DPPH.

#### 2.7.2 In vivo anti-oxidant assay

The study protocol was approved by the institutional animal ethical committee (IAEC), MDU, Rohtak, vide reference number 1767/RE/S/14/CPCSEA/CAH/76–85 dated 26–02-2021. The animals (Swiss albino mice) were procured from the Lala Lajpat Rai University of Veterinary and Animal Sciences (LLRU-VAS), Hisar, India. All the mice were provided the dried feed and water *ad-libitum* and maintained as per the provisions of the committee for the purpose of control and supervision of experiments on animals (CPCSEA). The small animals (25–30 g) were divided into five groups (n = 5) and acclimatized to lab conditions for ten days. The first group received the normal saline and termed as control, the second group was injected with scopolamine at a dose of 0.4 mg/kg through the intra-peritoneally (i.p.) route (negative control), the third group (positive control) received the donepezil (3 mg/kg, ip.) and the fourth and fifth groups were termed as test groups and mice were given the extracts in an oral dose of 200 mg/kg for two weeks. On the 15th day, animals of the test groups were also given scopolamine (0.4 mg/kg i.p.) before evaluating various biochemical parameters. Various *in-vivo* anti-oxidant parameters such as reduced glutathione, catalase, nitric oxide and superoxide dismutase (SOD) were determined [28]. For this, the whole brain was isolated from the animals of each group and washed with normal saline. The brain samples were homogenized with phosphate buffer (pH 7.4) and centrifuged at 10,000 rpm for 15min. The homogenate was used to determine various biochemical parameters as per the established procedures. The concentration of reduced glutathione was determined by the Ellman’s method. To describe briefly, equal quantities of brain homogenates and trichloroacetic acid (10 %) were mixed and centrifuged for 15 min. The supernatant was separated and mixed with phosphate buffer (pH 8.4), 5,5′-dithio*bis* (2-nitrobenzoic acid, (DTNB) and distilled water. The absorbance of the mixture was recorded in UV–Visible spectrophotometer at λ_max_ of 412 nm. The catalase activity was evaluated by Aebi’s method [29]. To carry out this, 100 μL of the brain homogenate, phosphate buffer (0.1 mM, pH 7.4) and hydrogen peroxide (30 mM) were mixed and the reaction mixture was analyzed at λ_max_ 240 nm in an UV spectrophotometer. Changes in absorbance were noted and expressed as nmol of H_2_O_2_ consumed/min/mg/protein. The nitric oxide (NO) contents in the brain homogenates were evaluated using the Griess reagent and expressed as µmol/mg of tissue [30]. Briefly, the homogenate was mixed with phosphate buffer and sodium nitroprusside and incubated at 25 °C. After 3 h, the Griess reagent (0.5 mL) was mixed with an equal quantity of the solution and again incubated for 30 min at room temperature. Due to the binding of nitrite with the Griess reagent, the colored azo derivatives (chromophores) are formed and can be measured by a spectrophotometer at λ_max_ of 540 nm. The superoxide dismutase (SOD) concentration was also estimated in the different samples of brain homogenates by a spectrophotometric method [31]. To perform this, *n*-butanol was mixed in brain samples with continuous stirring. The mixture was incubated at room temperature for 15 min and then centrifuged to separate the butanol layer. The absorbance of the solution was measured (λ_max_ 560 nm) to calculate the SOD concentration.

#### 2.7.3 Evaluation of the AChE inhibitory potential

The well-established Ellman’s method was employed to evaluate the AChE inhibitory potential (*in vitro* and *in vivo*) of the different extracts [32]. Briefly, the extracts were dissolved at different concentrations in Tris HCl (50 mM). The AChE enzyme (for *in vitro* assays) and brain homogenates (for *in vivo* assays) were mixed with phosphate buffer (0.1 M, pH 8) and dithio-*bis*-nitrobenzoic acid (DTNB). The reaction mixture was incubated for 15 min at room temperature. The substrate, acetylcholine iodide, was added to the mixture before analyzing it with an UV–Visible spectrophotometer at λ_max_ of 412 nm.

### 2.8 Statistical analysis

All the procedures were conducted in triplicate and results expressed as mean ± S.D. Also, the significance of the data was evaluated by variance analysis (ANOVA) followed by Tukey test using the Graphpad Prism 9.0 software. The probability to get the observed results, *p* < 0.05 was considered as statistically significant.

## 3 Results and Discussion

Study of the literature reported that there is a tremendous ambiguity in the taxonomic nomenclature of the selected medicinal plant. *N. jatamansi* is massively confused with *Valeriana jatamansi* due to overlapping of trade or vernacular names. Mabberlerie and Notlie [33] have indicated that scientists are still using the wrong citation of this herb in their papers and there is an urgent need to address this problem. Moreover, as per the reports of the National Medicinal Plant Board of India, this plant is mostly collected from wild sources by untrained personnel. Hence, to begin with research on the procured sample of *Jatamansi*, it was identified and authenticated as *Nardostachys jatamansi* (D. Don) DC by Dr. Sunita Garg (Principal Scientist) of the CSIR-NISCAIR (NISCAIR/RHMD/Consult/2018/3157–06-2 dated 19/03/2018), based on detailed scrutiny of literature and matching the morphological features with the authentic sample deposited in the Raw Material Herbarium and Museum of Delhi. The selected plant displays great therapeutic potential and its properties are also mentioned in the ancient Indian literature for the treatment of various ailments. It is traditionally used in the prevention of mental illness and improvement of learning and memory. Several reports have indicated that the phenolics and flavonoids present in this herb are responsible for its anti-oxidant potential [34], [35]. It is among the top traded medicinal plants around the Globe. Moreover, about 25 commercial Ayurvedic formulations are available in the market, which signifies the importance of this herb for the prevention or the treatment of various diseases. The herb is generally extracted by conventional extraction methods (percolation or maceration) by manufacturing companies to meet the demand of extracts in India and other countries. Nevertheless, regarding the limitations of traditional methods such as high time and solvent consumption, the paradigm has now shifted toward non-conventional extraction procedures. The present research aimed at establishing an UAE method for the extraction of NJ roots as well as the optimization of various extraction parameters, so that extracts can be prepared in a faster way by adopting the green approach.

Literature indicated that in >60 % of cases, roots of *N. jatamansi* (D. Don) DC are extracted by the percolation method using alcohol or water as solvents [19]. Therefore, we also prepared extracts of this plant by the conventional percolation method using hydroalcoholic solvent (20:80) in the soxhlet apparatus. Moreover, the extraction of plant actives, including total phenolics is generally carried out with polar solvents (ethanol or water) [36]. Hence, we employed the hydroalcoholic solvent for extraction by the soxhlet method and the yield of extracts, TPC and TFC were calculated to be 6.20 ± 0.81 w/w, 38.56 ± 1.04 mg GAE/g, 28.33 ± 0.94 mg RUE/g, respectively.

Nowadays, the UAE is extensively used for the improved and efficient extraction of crude plant samples compared to traditional methods. It is considered not only to be a cost-effective method, but it also expedites the extraction process in an environmentally-friendly manner. Vibrations caused by the sound waves produce small jets inside the solvent and create microbubbles, which grow in size and burst near the surface of the particles to bring out the cavitation effect. This process leads to enhanced migration of the solvents inside the plant samples, improved solubilization of extractable matter and efficient migration of extracts into the surrounding medium. Extraction using UAE is generally affected by numerous variables such as sonication frequency, process temperature, solvent nature and its viscosity, process duration, solvent concentration and pulse of sound waves generation, etc. Therefore, some preliminary experiments and an extensive literature survey were conducted to select the process variables and their range for optimization. As a result, a sonication frequency of 24 KHz, a processing temperature of 70 °C and ethanol as solvent were selected for the UAE of NJ roots. Also, the maximum exposure to heating in solution mixtures can be prevented by interval pulse modulation to enhance the experimental results. Therefore, a pulse of 0.5 *sec* at low intensity was selected on basis of the results of preliminary experiments. The optimization of the process can confirm the reliability, reproducibility and effectiveness of the extraction process in UAE. Hence, the central composite design (CCD) coupled with response surface methodology (RSM) was applied for the optimization of various process variables like duration of sonication (X_1_, 10–30 min). The viscosity and the volume of the solvent with respect to plant particles, significantly affected the movement of sound waves in the medium. Therefore, the volume of added water needs to be increased to reduce the viscosity of ethanol (concentration of solvent, X_2_, 60–80 %). The ratio of the solvent to the plant sample volume of solvent, X_3_, (15–25 mL/g) was also optimized for better extraction efficiency. A total of twenty experiments were conducted which represents six axial points, eight factorial points and six central points.

### 3.1 Adjustment of the developed model

The different experimental conditions followed, and the actual and predicted values of the different dependent variables (EY, TPC and TFC) are presented in Table 1. Data were analyzed and the statistical significance of the obtained results was predicted by applying variance analysis (Table 2).

Polynomial regression equations were also generated (**Eq**. 2,3,4) to indicate the magnitude of the effect of the different process variables on the selected responses (Y_1_, Y_2_, Y_3_).

(2) $$Y_{1}=77.84+0.46X_{1}+1.87X_{2}+0.012X_{3}+0.85X_{1}X_{2}+2.21X_{1}X_{3}+2.20X_{2}X_{3}+43.88X_{1}^{2}+33.16X_{2}^{2}+1.88X_{3}^{2}$$

(2) $$Y_{2}=2613.66+8.58X_{1}+174.07X_{2}+77.72X_{3}+24.61X_{1}X_{2}+{74.60X}_{1}X_{3}+100.47X_{2}X_{3}+995.56X_{1}^{2}+1368.14X_{2}^{2}+{47.01X}_{3}^{2}$$

(3) $$Y_{3}=1922.38+1.19X_{1}+8.14X_{2}+91.78X_{3}+1.53X_{1}X_{2}+0.36X_{1}X_{3}+9.03X_{2}X_{3}+824.95X_{1}^{2}+1116.87X_{2}^{2}+144.29X_{3}^{2}$$

**Fig. 1:**
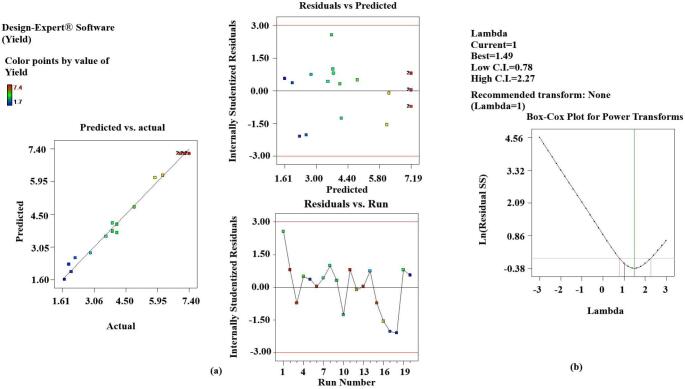
Diagnostic plot for EY **a.** Actual results versus predicted results and residuals against predicted and actual runs; **b**. Box-cox plot for power transform.

**Fig. 2:**
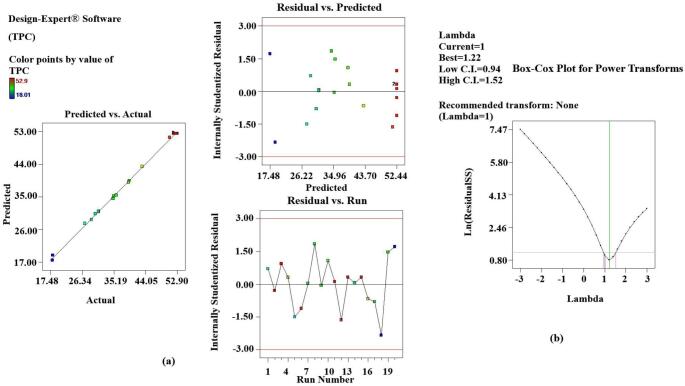
Diagnostic plot for TPC **a.** Actual results versus predicted results and residuals against predicted and actual runs; **b**. Box-cox plot for power transform.

**Fig. 3:**
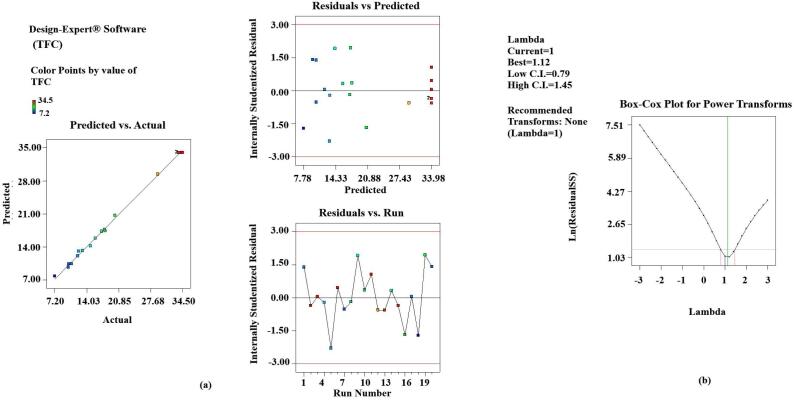
Diagnostic plot for TFC, **a.** Actual results versus predicted results and residuals against predicted and actual runs; **b**. Box-cox plot for power transform.

Statistical analysis of the data confirmed the adequate functionality of the results by the developed model (p < 0.001). Results of the experiments also indicated that the linear and interactive effects of independent variables (X_1_, X_2_ and X_3_) on dependent variables were also significant (p < 0.05) except for TFC (X_1_ and X_1_X_3_ > 0.05). The values of the correlation coefficient R^2^ for all the responses were found to be close to one, which suggests that most of the variations can be explained by the developed model. Also, the closeness of R^2^ and the predicted R^2^ to each other confirmed that the variables (dependent and independent) were well correlated. The diagnostic curves also confirmed the suitability of the developed model and the obtained results. The graphs between the predicted results and the actual results, the residuals against the actual runs and the predicted runs, indicated that all the results obtained were within the confidence limit of the predicted ones Fig. 1 (Fig. 1.a, 2.a, 3.a). The box-cox plots indicated that the λ values were close to those predicted and no power transform was suggested by the software (Fig. 1.b., 2.b, 3.b).Fig. 2.Fig. 3..

**Fig. 4:**
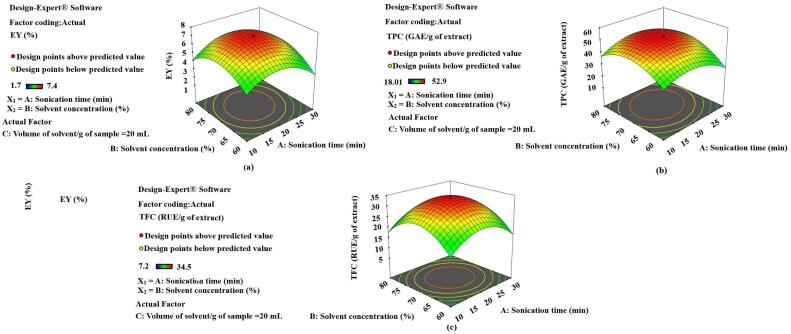
3-D diagrams indicating the effect of different variables (X_1_,X_2_) on EY, TPC and TFC.

The model developed for the different variables and responses also predicted the low coefficient of variance and a high adequate precision (see Table 2), which illustrates the accuracy and the consistency in the obtained results. Moreover, the non-significance of the pure error in the developed model was exhibited by absence of the adjustment value (p > 0.05).

### 3.2 Response surface analysis in UAE

**Fig. 5:**
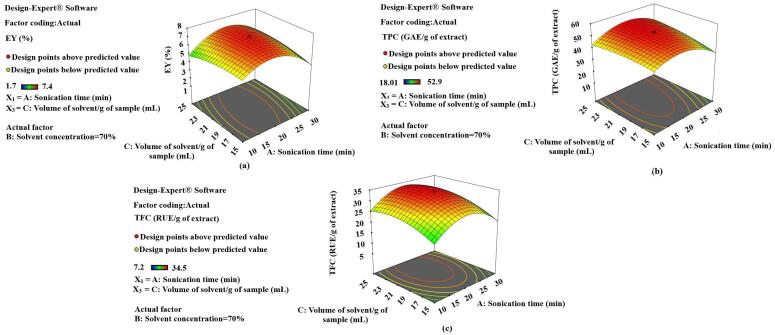
3-D diagrams indicating the effect of different variables (X_1_, X_3_) on EY, TPC and TFC.

**Fig. 6:**
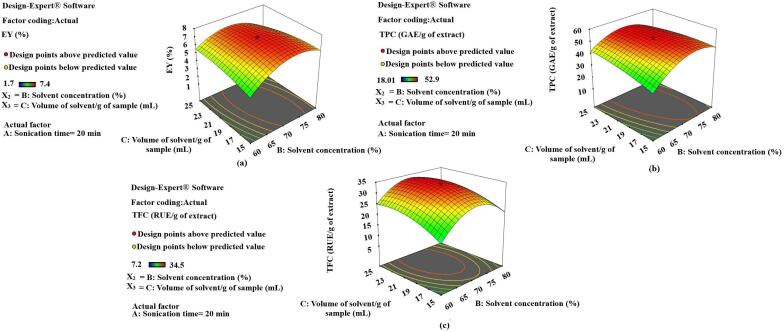
3-D diagrams indicating the effect of different variables (X_2_, X_3_) on EY, TPC and TFC.

The effect of selected variables on different responses has been figured in different 3-D diagrams (**Fig. 4, 5, 6**). Various factors influence the yield of extract and plant active contents in UAE. Some process parameters like temperature during extraction (70 °C), frequency of sound waves (24 KHz) and impulse (0.5 *sec*) have been fixed, based on some preliminary experiments. The sonication time (X_1_), the concentration of the solvent (X_2_) and the volume of the solvent (X_3_) with respect to the plant samples have also been optimized for EY, TPC and TFC in SXNJ and OUNJ extracts. From Fig. 4, one can analyze the effect of different sonication times and solvent concentrations at a fixed ratio of solvent to samples. It has been observed that when increasing the time of sonication, 10 to 20 min and the solvent concentration from 60 to 70 %, there was a significant rise in the EY, TPC and TFC (p < 0.01). Enhancement in the yield as well as in the phenolic and the flavonoid contents could be attributed to an increased polarity of the solvent mixture, the target compounds being more soluble in polar solvents. Moreover, it has also been reported that sound waves travel better in polar solvents to produce the acoustic cavitation effect [37], [38]. However, after that point, no more improvement in the response was observed, instead it declined sharply. Reduction in the yields of TPC and TFC could be due to an oxidation of plant metabolites by free ions generated by the dissociation of a higher amount of water molecules present as solvent [39].

Also, the duration of the exposure to sound waves affects the yields of the constituents. The response surface 3-D diagrams (**Fig. 5**) also indicated the effect of sonication time along with solvent concentration and volume of solvent/g of samples. A review of the literature suggests that optimization of the sonication time is highly required for better yields of phytoconstituents. Sonication times of 5–60 min are generally applied depending on the cellular structure of the plant sample and the resistance to heat mass transfer [2]. For example, hard nuts of *Areca catechu* were extracted for 50 min whereas a sonication time of only 5.9 min was sufficient for Thymus leaf extraction by UAE [40], [41]. In the present study, a range of 10–30 min for optimization of the sonication time was chosen by conducting some preliminary experiments. The exact optimum was selected by analyzing the results of various experiments conducted under different sets of conditions. These indicated that EY, TPC and TFC enhanced significantly with a rise in the sonication time but after certain periods, they decreased abruptly. These results are in agreement with previous ones regarding the extraction of phenolic compounds from olive leaves, which was found to be optimum at 20 min of UAE and thereafter decreasing with additional sonication of the samples [42]. This suggests that sonication times exceeding an optimum could lead to a reduction in yields of plant actives due to the shift of mass transfers inside the cells of the plant sample. Also, exposure to sound waves for longer than essential is not cost-effective and could lead to secondary metabolite deterioration due to the heat produced by the ultrasonic probe [5], [43].

The volume of the solvent required per g of plant sample is also an important variable in the optimization of UAE of herbs. The 3-D diagram presented in Fig. 6 indicates the effect of the volume of the solvent along with sonication time and solvent concentration on various dependent variables (EY, TPC, TFC). The optimum volume of the solvent, near to 20 mL/g of sample, allowed the recovering of the highest yields. These results also matched with past researchers’ observations reporting optimized solvent to solid ratios of 15:1 and 25:1 for the UAE of *Hypericum perforatum* and *Hemerocallis citrinabaroni*, respectively [44], [45]. A suitable amount of solvent is always required as it helps in the solubilization of non-polar metabolites and also acts as a medium for the traveling of the sound waves. Nevertheless, after exceeding a certain ratio, this will hamper the sonication process by causing a disturbance in the movement of radiations. Moreover, higher concentrations of solute particles could also increase the operation cost as more power is required for producing the cavitation effect [46].

### 3.3 Validation of the developed model

**Fig. 7:**
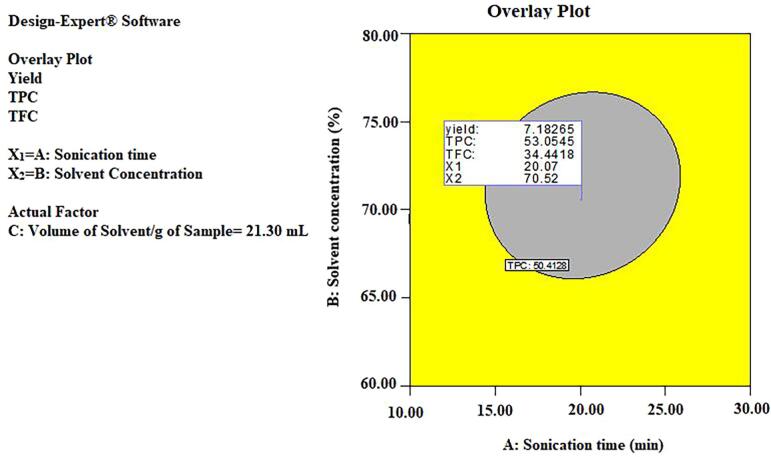
Overlay plot for the point prediction of optimized extraction conditions.

The developed model was also used to maximize the experimental conditions by a numerical optimization approach and prediction of the sonication time (20.10 min), the solvent concentration (70.46 %) and the volume of the solvent (21.43 mL) for the extraction of the selected plant by the sonication method. An overlay plot was also generated for the point prediction of extraction conditions and responses (Fig. 7). The experiments were also conducted at the predicted optimized conditions and the EY, TPC and TFC were calculated to be 7.85 ± 0.62 % (w/w), 52.94 ± 0.12 GAE/g, and 33.78 ± 0.15 RUE/g, respectively. All the results were within the 95 % confidence limit and confirmed the accuracy and predictability of the developed model for the selected variables.

It has been found that the volume of solvent/g of solid was optimized to 20:1 in UAE extracts as compared to 30:1 in SXNJ extracts, which did not only efficiently improve the yield of extract, TPC and TFC (p < 0.05) but also reduced the extraction time from 13 h in SXNJ to approximately 20 min in OUNJ. Similar results were also reported in the past, where the UAE was found to enhance the yields of TPC and TFC from the flowers of *Jatropha integerrima* as compared to conventional methods at optimized conditions [47]. Also, the seeds of *Eurylae ferox* were efficiently extracted within 21 min (as suggested by the CCD) of the sonication process [48]. From these results, we can suggest that the CCD coupled RSM could be successfully employed to optimize the extraction conditions in UAE for the selected herb.

### 3.4 Scanning electron microscopy (SEM)

**Fig. 8:**
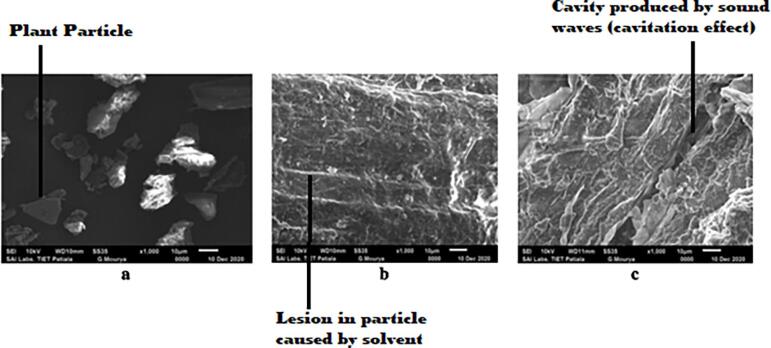
Microscopical pictures (1000x) of the plant samples (a) Untreated (b) after extraction by CPM (c) after UAE.

Microscopical alterations in the morphology of the plant samples which affect the different extraction processes, can be analyzed by using scanning electron microscopy (SEM). The magnified image (1000 × ) of untreated and processed (after extraction) plant samples can be visualized in Fig. 8. Before extraction, the powder of the herb revealed different particles without any striations on the surface (Fig. 8 a). After extraction by the conventional percolation method, the particles became flat and lesions were formed on their surface due to softening by the solvent (Fig. 8**b**). Herb processing with UAE brought out significant structural changes in the plant samples (Fig. 8**c**). Due to the mechanical effect of the microjets produced by sound waves in the solution, cavities formed on the surface of the solid particles. Moreover, the intensity of the cavitation enhanced for the solvents with low vapor pressure and surface tension [49]. Hence, binary mixtures of polar solvents, ethanol and water, with vapor pressures of 0.08 and 0.03 atm, respectively, further helped in inducing the cavitation effect. Also, heating of the solution due to the sonication process caused the crumbling of the cell surface [50], [51]. Based on SEM studies of the different plant samples, it can be deduced that sound waves significantly alter the cellular structure of the particles and thus could be helpful in improved and fast movement of the solvent to bring out a better extraction of plant actives. These results are also concomitant with previous studies showing that SEM analysis of Soybean flakes after the UAE confirmed the microfractures formed in the samples [39].

### 3.5 GC–MS analysis

**Fig. 9:**
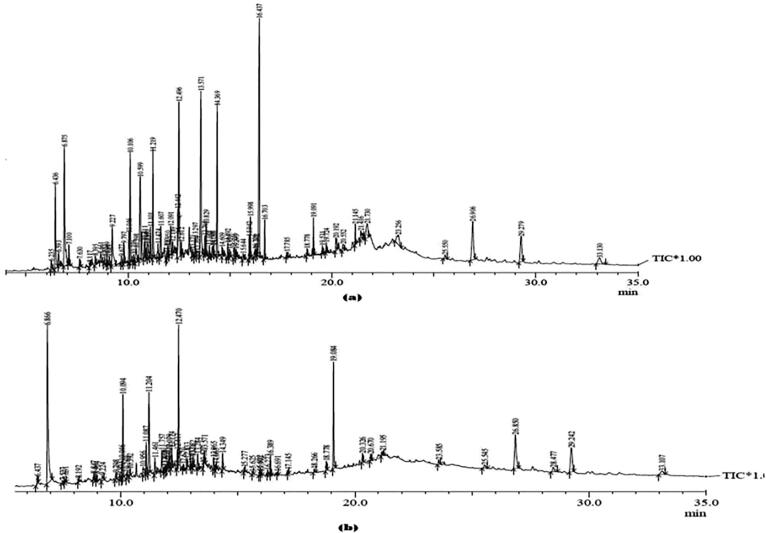
GC–MS chromatograms of different extracts (a) SXNJ (b) OUNJ. This figure should be read concomitantly with Table 3.

Separation and identification of the various constituents present in the different extracts of the roots of NJ were carried out using GC–MS analysis. The chromatogram indicating the peaks of the different plant actives is shown in Fig. 9. Moreover, the retention times (R_T_) of the various phytoconstituents along with their relative % area, are presented in Table 3. Analysis of the SXNJ and OUNJ extracts provided an idea about the presence of various sesquiterpenes, steroids and ester compounds in both extracts. The yields of therapeutically important sesquiterpenes such as jatamansone (↑91.8 %), spirojatamaol (↑42.1 %), globulol (↑130.4 %) and the steroid-like sitosterol (↑84.6 %) were significantly enhanced in the OUNJ extracts as compared to the SXNJ ones. Also, some compounds like the nootkatone (R_T_ 12.67), nardostachnol (R_T_ 11.08), stigma-3,5-diene-7-one (R_T_ 28.47) and pentafluoro-propionic acid dodecyl ester (R_T_ 10.04) were only recovered in OUNJ. GC–MS analysis depicted that the concentration of all plant actives was significantly improved, these results being in agreement with previous studies showing that UAE of *Enicostema littorate* and *Momordica charantia* improves the yield of secondary metabolites like swertiamarin (↑2.8-fold) and charantin (↑2.74-fold) as compared to conventional extraction methods [52], [53]. Heating of the plant samples and the cavitation effect produced by the sonication process at the optimized conditions of UAE could be responsible for the improved yields of plant actives [2].

**Table 3:** Different phytoconstituents identified in GC–MS analysis of SXNJ and OUNJ extracts of the selected plant along with their retention times (R_T_) and % area.

	SXNJ		OUNJ	
Chemical Constituents	R_T_ (min)	Area (%)	R_T_ (min)	Area (%)
**Spirojatamol**	**10.106**	**4.13**	**10.094**	**5.87**
**Nardostachnol**	**–**	**–**	**11.087**	**2.38**
**Jatamansone**	**11.219**	**3.97**	**11.204**	**7.57**
Valerenal	11.607	1.45	11.937	0.36
**Globulol**	**12.496**	**3.85**	**12.470**	**8.87**
**Nootkatone**	**–**	**–**	**12.670**	**0.44**
Ethyl oleate	15.998	1.12	15.992	0.22
Octadecanoic acid, ethyl ester	16.228	0.40	15.992	0.20
Stigmasta-5,22-dien-3-ol,acetate, −3-beta	–	–	23.585	0.57
**Gamma-sitosterol**	**26.906**	**3.83**	**26.850**	**7.07**
**Gamma-sitostenone**	**29.279**	**2.56**	**29.242**	**5.59**
Stigmastane-3,6-dione,5-alpha	33.130	1.12	33.107	1.18
Cyclopentane, 1-(3-methylbutyl)	9.227	2.99	9.224	0.78
**Aromadendrene, dehydro**	**11.101**	**1.21**	**11.757**	**2.13**
Naphthalene,1,2,3,5,6,7,8,8A-octahydro-1,8A-dimethyl-7-(1-methylethenyl)	8.969	0.34	8.964	0.42
2,11-dioxatetracyclo(4,3,1,1(3,10,)0,(6,9))undec-4-ene,3,7,7,10-tetramethyl	11.474	0.71	11.461	1.55
Isovalencenal	12.189	0.51	12.174	1.58
Sinularene	12.612	0.39	–	–
Stigmasta-3,5-diene-7-one	–	–	28.477	2.26
Cyclopentaneacetaldehyde,2-formyl-3-methyl-alpha-methylene	6.436	3.68	6.437	0.66
**2-(1-Methylcyclopropyl)aniline**	**6.875**	**6.36**	**6.866**	**20.82**
6-isopropenyl-4,8A-dimethyl-3,5,6,8,8A-hexahydro-1*H*-naphthalene-2-one	12.091	1.72	12.079	1.22
Coumarin-6-ol,3,4-dihydro-4,4,5,7-tetramethyl-methylsulfate(ester)	–	–	12.417	0.92
Ergost-5-en-3-ol, 3-beta	25.550	0.32	25.545	0.70
Stigmasta-3,5-diene	–	–	28.477	0.97
Pentafluropropionic acid, dodecyl ester	–	–	10.046	1.29

### 3.6 anti-oxidant potential of the extracts

**Fig. 10:**
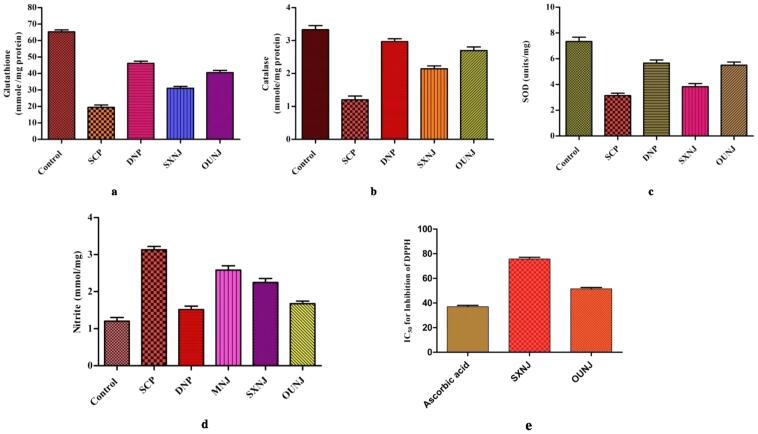
*In vivo* biochemical estimation of different anti-oxidant stress parameters (a) reduced glutathione (b) catalase (c) SOD (d) nitrite content in various groups (Control; SCP- Scopolamine induced group (negative control); DNP- Donepezil treated (positive control); SXNJ- test group (soxhlet extracts of *N. jatamansi*); OUNJ- test group (optimized extract formed by UAE of *N. jatamansi*) and (e) IC_50_ for scavenging of DPPH radicals (*in vitro*).

Oxidative stress and reactive oxygen species (ROS) are held responsible for numerous diseases in humans including neurodegeneration, Alzheimer disease and inflammation, etc. [54], [55], [56], [57], [58]. Extracts of *N. jatamansi* are also included in a number of marketed formulations for the prevention and treatment of such diseases [14], [17]. Thus, the anti-oxidant potential of the different extracts of *N. jatamansi* (D. Don) DC, SXNJ and OUNJ, were evaluated by *in-vitro* and *in-vivo* assays. The DPPH assay was used to evaluate the anti-oxidant potential of the extracts under *in vitro* conditions. Scavenging of free radicals of DPPH by antioxidants present in the extracts faded the violet color of the solution. Also, alteration in color intensity was proportional to antioxidants concentration in the test solution [59]. In the present case, we observed that the IC_50_ to curb the free radicals was significantly reduced (p < 0.05) in the OUNJ extracts as compared to the SXNJ ones (Fig. 10**e**). Moreover, the extracts were also evaluated for their *in vivo* anti-oxidant potential. Scopolamine was used to increase the oxidative stress level in experimental animals and it was found that enhanced stress significantly altered various biochemical parameters such as glutathione, catalase, SOD and nitrite in the negative control group (p < 0.01) (Fig. 10**a**,10b,10c,10d). It has been observed that changes in the concentration of glutathione, catalase and nitrite in the OUNJ group were significantly different as compared to the negative control group (p < 0.01) as well as the SXNJ group animals (p < 0.05). Data indicated that SXNJ extracts altered the selected biochemical parameters, as well (p < 0.05). Thus, interpretation of the results regarding the anti-oxidant assays confirmed the protective effect of OUNJ extracts as well as SXNJ extracts against scopolamine-induced oxidative stress. It has also been observed that the antioxidant protective effect was significantly improved in the optimized extracts as compared to those obtained by conventional percolation (SXNJ). The enhanced protective effect of the OUNJ extracts could be attributed to increased phenolic and flavonoid contents in those extracts. Phenolic or flavonoid compounds are proven to possess anti-oxidant properties by donating hydrogen atoms to free radicals [60], [61]. It was also reported that the quenching of free radicals was improved with an increased concentration of such metabolites [62]. Literature suggests that along with phenolic or flavonoid compounds, sesquiterpenes also possess anti-oxidant properties [63]. The globulol and nootkatone sesquiterpenes found in the NJ extracts were also found to scavenge free radicals in various past studies [64], [65]. In the present work, GC–MS analyses of the extracts proved that the concentrations of various sesquiterpenes like jatamansone, spirojatamol, globulol, nootkatone were significantly increased in the OUNJ extracts as compared to the SXNJ ones. The observed improved protection against the oxidative stress by OUNJ extracts could thus be attributed to the enhanced concentration of these various sesquiterpenes along with TPC and TFC.

### 3.7 AChE inhibitory potential of the different extracts

**Fig. 11:**
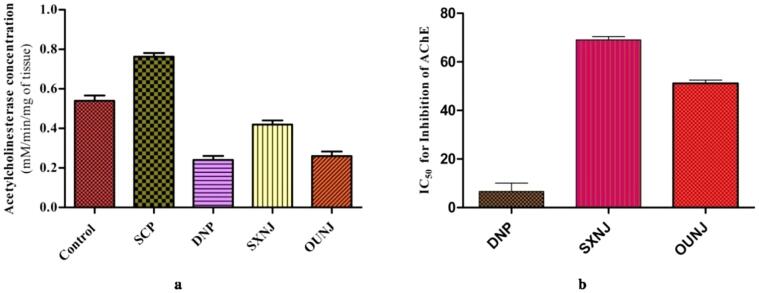
(a) AChE concentrations in brain tissues (*in vivo*) (Control; SCP- Scopolamine induced group (negative control); DNP- Donepezil treated (positive control); SXNJ- test group (soxhlet extracts of *N. jatamansi*); OUNJ- test group (optimized extract formed by UAE of *N. jatamansi*) (b) IC_50_ for AChE inhibition in µg/mL (*in vitro*).

*N. jatamansi* root extracts possess a remarkable pharmacological potential useful in various neurodegenerative ailments including Alzheimer disease [17], [66]. In the past, the NJ extracts were also reported to inhibit the AChE enzyme, which plays a fundamental role in Alzheimer disease [67]. The importance of AChE inhibitors has been well established in attenuating Alzheimer disease symptoms [14], [68], [69], [70]. Thus, the effect of the optimized extracts along with conventional extracts on the AChE (*in vitro* and *in vivo*) has been studied in the present research. *In vitro* assays indicated that the IC_50_ for AChE inhibition was significantly reduced from 69.11 ± 2.38 µg/mL in the SXNJ extracts to 51.15 ± 1.28 µg/mL in the OUNJ ones (p < 0.05) (Fig. 11**b**). Moreover, analysis of AChE in brain homogenates of experimental animals confirmed that scopolamine significantly enhanced its concentration (Fig. 11**a**). Treatment with the optimized extract (OUNJ) significantly reduced AChE concentrations as compared to the negative control (p < 0.01) and the SXNJ extract (p < 0.05). As well, the extract obtained by the conventional method (SXNJ) significantly inhibited AChE at the test doses. The present results could be attributed to an improved concentration of secondary metabolites especially that of sesquiterpenes, like jatamansone, spirojatamaol, globulol, etc in the OUNJ extracts. Past studies proved that cholinergic transmission can be ameliorated by various sesquiterpenes through AChE inhibition and thus could be employed for the management of Alzheimer disease [14]. Moreover, the concentration of nootkatone increased in the OUNJ extracts and it has been reported that this plant active could play a significant role in reduction of the neuro-inflammation associated with Alzheimer disease by altering cholinergic transmission, oxidative stress and expression of the transcription factor NF- κB-*P*65 [65]. Various researchers have also shown that the phenolic content from herbs could be linked to free radical scavenging in brain tissues and can improve the cognitive function of experimental animals [71], [72]. Thus, we can say that improvement in the concentration of various plant actives reported in this work, is responsible for the significant alteration in the anti-oxidant as well as the AChE inhibition potential of the optimized extracts.

## 4 Conclusion

*Nardostachys jatamansi* (D. Don) DC is a potential medicinal herb, utilized in substantial ayurvedic formulations for the prevention and treatment of various ailments. In past years, the commercial significance of this medicinal plant had also increased potentially and thus the demand for herb extracts is enhanced in India and abroad. Conventional methods of extraction alone cannot meet the demand of the industry. Moreover, traditional extraction methods carry the limitations of not being environmentally friendly as well as being time-consuming. Hence, the paradigm has to be shifted toward non-conventional techniques such as UAE for improving extraction in less time and also accounting for a green approach. In the present research, the UAE method has been developed and optimized for the extraction of NJ roots by using a response surface methodology. It has been found that extracts obtained at optimized conditions of UAE not only possess similar metabolites (compared to a conventional extract) but the quality of the process was also improved in terms of concentration of the plant actives. Moreover, the time of extraction as well as the pharmacological potential (anti-oxidant and AChE inhibition activities), were also improved in the optimized extracts. To conclude with, we can suggest that after suitable improvement of the developed method, application at an industrial scale can be considered to meet the rising demand of extracts of that plant.

## Declaration of Competing Interest

The authors declare that they have no known competing financial interests or personal relationships that could have appeared to influence the work reported in this paper.

## Data Availability

Data will be made available on request.
